# Emerging Japanese Encephalitis Virus Genotype V in Republic of Korea

**DOI:** 10.4014/jmb.2207.07002

**Published:** 2022-07-25

**Authors:** Ah-Ra Lee, Jae Min Song, Sang-Uk Seo

**Affiliations:** 1 Department of Biomedicine and Health Sciences, Department of Microbiology, College of Medicine, The Catholic University of Korea, Seoul 06591, Republic of Korea; 2 School of Biopharmaceutical and Medical Sciences, Sungshin Women’s University, Seoul 01133, Republic of Korea

**Keywords:** Japanese encephalitis virus, genotype, Republic of Korea, vaccine

## Abstract

Japanese encephalitis (JE) is a vaccine-preventable mosquito-borne disease caused by infection with the Japanese encephalitis virus (JEV). JEV has five genotypes, including genotype V (GV), which is considered ancestral to the other genotypes. The first GV strain, GV Muar, was isolated from a Malayan patient in 1952 and GV did not reappear for 57 years until GV XZ0934 was isolated from a mosquito sample in China. Since 2010, 21 GV strains have been identified in Republic of Korea (ROK). Both GV Muar and GV XZ0934 are more pathogenic than other GI/GIII strains and are serologically distinct. However, because the ROK’s GV strains have not been experimentally tested, their characteristics are not known. Characterization of the ROK’s isolates is needed to enable development of effective GV strain-based vaccines to protect against GV infections.

## Introduction

Japanese encephalitis virus (JEV), a member of the Flaviviridae family, is an enveloped positive-sense RNA virus that can cause viral meningoencephalitis [1, 2]. Japanese encephalitis (JE) is a prevalent infectious disease with about 67,900 cases annually in 24 Southeast Asian countries and in the Western Pacific region [3]. JEV infection is asymptomatic in more than 99% of cases; however, once JEV-infected patients develop encephalitis, the case-fatality rate can be up to 30% [4]. Furthermore, 30%–50% of survivors have neurologic and psychiatric sequelae, making JE a particularly important public health problem if uncontrolled. Even though there are multiple effective vaccines against JEV [5], it remains a major pathogen due to its serious neuropathogenicity. JEV is a zoonotic mosquito-borne pathogen and humans are its dead-end host [6]. Continuous transmission of JEV from amplifying host to humans increases the possibility of emerging epidemics by new variants that facilitate spread of disease as seen in other mosquito-borne viral infections [7]. *Culex* (*Cx.*) species are potent primary vectors that transmit JEV in Republic of Korea (ROK) but other mosquitoes such as Aedes species can also carry the virus [8]. As different mosquito species have distinct vector competence for different JEV strains, changes in vector composition and distribution may also affect the circulating strains.

The first case of JE in ROK was reported in 1947 and JE remains endemic in ROK [9]. JE outbreaks have been greatly reduced since 1984 when a nationwide immunization program was implemented, confirming the efficient prophylactic efficacy of vaccine against JEV infection [10]. However, human JE cases are continuously detected in ROK and annual cases have clearly increased since 2010 [10]. The annual mean number of JE cases was 3.7 ± 3.0 in 2001–2009 and 21.7 ± 11.3 in 2010–2019. Analysis is needed to learn why JE cases have risen in order to minimize the impact of future JE epidemics.

## Genotype V (GV) Strains

JEV is currently subdivided into five genotypes mainly based on premembrane (prM) and envelope (E) gene sequences [11, 12]. In 1952, a serologically distinct JEV, Muar, was isolated from the brain of a JE patient in Malaysia [13]. Based on a series of serologic studies using monoclonal antibodies, it was confirmed that Muar belongs to a separate antigenic group [14, 15]. Early phylogenetic analysis also determined the Muar as a distinct GV [12, 16]. An evolutionary study revealed that GV is the ancestor of other genotypes (*i.e.*, GI~GIV) [17]. A follow-up study with phylogenetic simulation using whole genome sequence of all JEV genotypes also suggested that GV is nearest to the common ancestor and that GIV, GIII, GII, and GI evolved sequentially from GV [18].

Because GV and GIV strains are relatively older, it seems natural to find GIII and GI more frequently from recent isolates. Indeed, GIII strains were common in early JEV studies but were gradually replaced by GI strains in most endemic areas [19, 20]. In 2009, 57 years after isolation of the GV Muar, GV XZ0934 was isolated from *Cx. tritaeniorhynchus* in China [21]. When compared the whole genome between GV Muar and GV XZ0934, the similarity was 90.6% and 98.3% for nucleotide and amino acid sequences, respectively, reflecting a temporal gap between these two GV isolates [22].

**Fig. 1:**
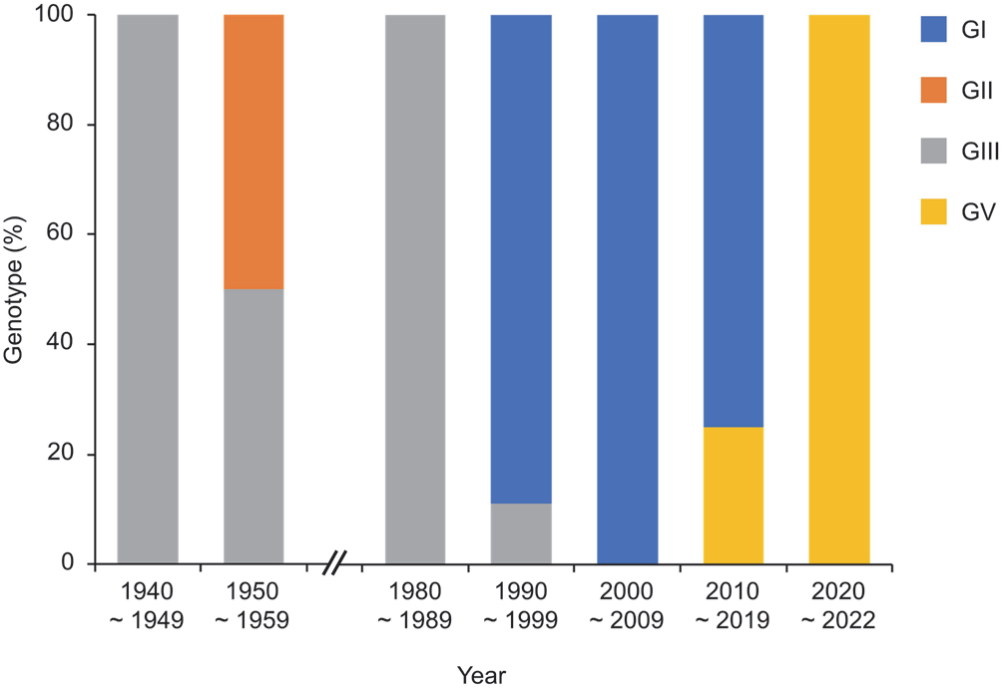
Frequency of reported Japanese encephalitis virus genotypes in Republic of Korea.

A year after the isolation of GV XZ0934, GV 10-1827 was isolated from a mosquito pool in ROK [23]. Since then, total 21 GV strains have been reported in ROK (Table 1). As in many other Asian JEV-endemic countries, GIII was prevalent before 1990 in ROK. Subsequently, GI dominated from the 1990s until 2010 when GV became the prevailing lineage after the isolation of GV 10-1827 (Fig. 1). Sequence analysis showed that genome identities of the E gene between GV 10-1827 and other genotypes (GI–GIV) are 77.3%–78.1% and amino acid identities are 90.4%–91.3% [23]. A subsequent study of 993 mosquito pools, found 6 GV strains [24]. In 2015, the first human GV isolate in ROK, GV K15P38, was obtained from the cerebrospinal fluid of a JE patient [25]. Sequence analysis showed that the entire open reading frame (ORF) sequence of GV K15P38 has 90.4% identity to the GV Muar, and the E gene sequence of GV K15P38 has 98.5%–99.8% similarity with other GV strains isolated from mosquitoes in ROK. The next study employed next-generation sequencing (NGS) techniques to analyze the mosquito virome [26]. The study analyzed 260 mosquito pools; 2 were positive for GV strains (GV A18.3208 and GV A18.3210). That study also retrospectively analyzed an NGS database and found another GV strain (GV 16-0830). The most recent JEV study in 2020 analyzed 4,953 mosquito pools. Of these, 7 pools were JEV-positive [27]. All 7 JEVs were GV. Two strains (GV Sangju-v1 and GV Sangju-v2) were isolated.

Overall, the epidemiologic data demonstrate a GI to GV shift in ROK but this observation has limitations because most of data are from mosquitoes. Only GV K15P38 has been isolated from human [25]. Further genotyping of human isolates is required to define the clinical dominance of GV strain in ROK.

## Mosquito Vectors in ROK

Vector surveillance is important to understand the epidemiology of vector-borne disease. *Cx. tritaeniorhynchus* is the primary vector found in most of JE-endemic areas [28]. Other mosquito species that can transmit JEV are *Cx. bitaeniorhynchus*, *Cx. pipiens*, and *Cx. orientalis*. Surveillance in ROK showed that *Cx. pipiens* was the most frequent mosquito species (37.8%), followed by *Cx. tritaeniorhynchus* (9.3%), *Cx. orientalis* (2.0%), and *Cx. bitaeniorhynchus* (<1%) [27]. Interestingly, when the first GV strains were isolated in ROK from 19 JEV-positive mosquito pools, GV 10-1827 was isolated from *Cx. bitaeniorhynchus*, whereas all 18 GI strains were isolated from *Cx. tritaeniorhynchus* [23]. Many of ROK’s GI and GIII strains were isolated from *Cx. tritaeniorhynchus* and *Cx. pipiens* [29]; however, only 1 of 21 ROK’s GV strains was isolated from *Cx. tritaeniorhynchus* (Table 1). Other studies reporting new GV strains in ROK showed that *Cx. bitaeniorhynchus*, *Cx. pipiens*, and *Culex orientalis* work as vectors for GV strains [24, 26, 27]. These results suggest that GV strains have different vector preference to other genotypes. While phylogeographic analysis clearly shows the transmission of GIII and GI in most endemic areas [20, 30-32], it is not clear why GV strains, starting in 2010, became the predominant JEV isolates in ROK. Further genomic, molecular, and epidemiologic characterization of GV strains are required for better understanding of GV prevalence in conjunction with vector studies.

## Characterization of GV Strains

Two earlier GV isolates, GV Muar and GV XZ0934, were tested for pathogenicity in mice. Mice infected with GV Muar had higher mortality rates than GI Mie/41-infected mice [33]. When several chimeric viruses were generated between GV Muar and GI Mie/41, E and prM proteins of GV Muar were responsible for increased virulence [34]. Specifically, histidine in position 123 of GV Muar E protein was most responsible for increased virulence. Also, GV XZ0934 was more neuropathogenic in a mouse model than GIII RP-9 [35]. In that study, mice were infected intraperitoneally either with GV XZ0934 or GIII RP-9 and GV XZ0934 had higher virulence. The chimeric GIII RP-9 virus, which has capsid, prM, and E proteins of GV XZ0934 gained more virulence which is comparable with GV XZ0934. Results of animal studies of both GV Muar and GV XZ0934 indicate that structural proteins are responsible for enhanced virulence.

Given that E protein works as a primary antigenic site and important virulence factor, the E protein seems responsible for many characteristics of GV strains. GV Muar has 22 signature amino acids in E protein and glutamine at position 327 was expected to play an important role for unique antigenicity as it is exposed on the receptor binding site [17]. When the E protein in ROK’s GV strains were compared with the E protein in GV Muar and GV XZ0934 (from Malaysia and China, respectively), 13 amino acid positions were variable [25]. Lysine at position 84 was conserved in all isolates from ROK; GV Muar and GV XZ093 have arginine in this position.

In vitro growth of JEV correlates with their virulence in mice [36]. As GV strains showed higher virulence than GI and GIII strains in mice, they were expected to grow better in cell lines. However, GV strains made smaller plaques than strains belonging to other genotypes [33, 35, 37]. GV Muar amplified more slowly than GI Mie/41 and GIII Beijing-1 in various cell lines. Histidine at position 166 of the NS2A protein was responsible for this phenotype [38]. This study indicates that both structural and non-structural proteins contribute to the pathology of GV strains.

The molecular basis for the pathogenesis of GV strains is still not well understood despite growing knowledge of its importance. Especially, virulence and other characteristics of ROK’s GV isolates have not been tested. Although previous studies suggested that GV Muar and GV XZ0934 have greater virulence than other JEV genotype strains, it is possible that the ROK’s GV strains have different pathogenicity and *in vitro* growth characteristics because of unique mutations [26].

## Vaccine against GV Strains

All current JEV vaccines are GIII-based inactivated or live-attenuated whole virus vaccines. As the genotypic shift from GIII to GI and GV are now frequent, it is important to evaluate the cross-genotype protection of GIII-based vaccines. Live-attenuated GIII SA14-14-2 vaccine was found to protect mice against lethal challenges with various GI strains [39]. Inactivated GIII SA14-14-2 vaccine also provided cross-genotype protection but was less efficient than the live-attenuated platform. Human serum samples from vaccinated donors exhibited similar neutralizing potential against GI, GII, GIII, and GIV strains [40].

Because JEV E protein plays a crucial role for neutralizing antibody response [41], phylogenetically most distinct GV strains should have least cross-reactivity to GIII-specific neutralizing antibodies. Indeed, in a mouse experiment that used inactivated GIII Beijing-1 vaccine, there was reduced protection against GV Muar [33]. Live GIII SA14-14-2 and inactivated GIII P3 vaccines were also less efficient in protecting mice from lethal GV XZ0934 challenge [37]. In an attempt to develop a lentiviral vector-based vaccine containing prM and E regions of GIII RP-9, the vaccine candidate elicited neutralizing antibody against GV XZ0934 but at a lower titer than against GIII and GI strains [42]. When sera of JE patients in Vietnam, the endemic country where no GV strain was reported, were subjected to 50% plaque reduction neutralization test (PRNT50), serum neutralized the GI Mie/41 more efficiently than GV Muar [43]. Overall, these studies suggest that anti-GIII antibodies raised by currently available commercial vaccines or by infection of non-GV JEV will induce less efficient immunity against GV strains than strains of other genotypes.

Some researchers have developed vaccine candidates containing GV Muar or GV XZ0934 antigens to maximize efficacy against GV strains. A virus-like particle (VLP) vaccine candidate that expresses GV Muar E protein elicited neutralizing antibody against GV Muar in rabbits and mice [44]. The rabbit immune sera of this VLP candidate vaccine also neutralized GIII Nakayama but was less efficient in neutralizing GIII Beijing-1. A sub-viral particle (SVP) presenting GV Muar E protein also elicited neutralizing antibody against GV Muar and other GV strains in mice, including isolates from ROK [45]. GV SVP vaccine induced increased PRNT_70_ titer when co-administered with inactivated GIII Beijing-1-based vaccine. One study employed a live-attenuated vaccine approach using an intertypic recombinant strain that contained E protein of GV XZ0934 in the GI Mie/41 backbone [46]. This recombinant JEV added 10 amino acids to the E protein to enable acquisition of more attenuated phenotypes. The vaccine protected mice from lethal challenge with GV Muar. The study results indicate that GV Muar and GV XZ0934 are serologically compatible and further suggest that ROK’s GV isolates may have similar compatibility.

## Conclusion

The impact of GV strains on public health is hard to predict based on current limited epidemiologic and pathophysiologic knowledge. Despite attempts to develop novel antiviral or drug repositioning for JEV, no drug is currently available and the vaccine remained the best option for the control of JE in humans [1, 47]. A high percentage of the population in a JE endemic area already possesses cross-reactive immunity against GV strains through vaccination and natural infection. However, GIII- or GI-specific immunity may be suboptimal against GV strains due to their distinct antigenicity. Also, acquired immunity gradually decreases from 1.5 to 8.5 years after vaccination and neutralizing antibody positivity is negatively correlated with age [48, 49]. The combination of waning antibody response and relatively lower vaccine coverage in adults and elderly populations (specifically those age 40 and older) make many people vulnerable to JE in China, Japan, and ROK [50-54]. If the assumption that GV will become the dominant lineage is correct, development of GV strain-based vaccines will be key for effective control of GV outbreaks. Vaccines can be developed in multivalent formulations to protect against co-circulating genotypes as this approach can induce stronger and broader neutralizing antibody responses [45]. Eventually, antivirals for JEV may enhance preparedness against newly emerging genotypes.

## Figures and Tables

**Table 1:** Frequency of reported Japanese encephalitis virus genotypes in Republic of Korea.

Year	GV strain	Locality	Host	Specimen	Gene	GenBank accession number	Reference
1952	Muar	Malaysia	Homo sapiens	Brain	Complete	HM596272	[13]
2009	XZ0934	Tibet, China	Mosquito	*Cx. tritaeniorhynchus*	Complete	JF915894	[22]
2010	10-1827	Daeseondong, Gyeonggi, ROK	Mosquito	*Cx. bitaeniorhynchus*	E NS5	JN587258 JN587243	[23]
2012	K12HC959	Hwacheon, ROK	Mosquito	*Cx. orientalis*	E	KJ420589	[24]
	K12AS1148	Ansan, ROK	Mosquito	*Cx. pipiens*	E	KJ420590	
	K12AS1151	Ansan, ROK	Mosquito	*Cx. orientalis*	E	KJ420591	
	K12YJ1174	Yeoju, ROK	Mosquito	*Cx. orientalis*	E (partial)	KJ420593	
	K12YJ1182	Yeoju, ROK	Mosquito	*Cx. orientalis*	E	KM496505	
					C	KJ420595	
					E (partial)	KJ420594	
	K12YJ1203	Yeoju, ROK	Mosquito	*Cx. orientalis*	E	KJ420592	
					C	KJ420596	
2013	K13GB57	Gyeongsan-si, ROK	Mosquito	*Cx. tritaeniorhynchus*	E	KM496503	GenBank
2015	K15P38	Gyeonggi-do, ROK	Homo sapiens	Cerebrospinal fluid	Complete	MK541529	[25]
					E	MF526903	
2016	16-0830	Yongsan, ROK	Mosquito	*Cx. orientalis*	Complete	MT568540	[26]
2018	A18.3208	Camp Hamphreys, ROK	Mosquito	*Cx. bitaeniorhynchus*	Complete	MT568539	
	A18.3210	Camp Hamphreys, ROK	Mosquito	*Cx. bitaeniorhynchus*	Complete	MT568538	
2020	Sangju-1	Sangju, ROK	Mosquito	*Cx. orientalis*	NS5	MZ868499	[27]
	Sangju-2	Sangju, ROK	Mosquito	*Cx. orientalis*	NS5	MZ868500	
	Sangju-3	Sangju, ROK	Mosquito	*Cx. orientalis*	NS5	MZ868501	
	Sangju-4	Sangju, ROK	Mosquito	*Cx. orientalis*	NS5	MZ868502	
	Sangju-5	Sangju, ROK	Mosquito	*Cx. orientalis*	NS5	MZ868503	
	Sangju-6	Sangju, ROK	Mosquito	*Cx. orientalis*	NS5	MZ868504	
	Sangju-7	Sangju, ROK	Mosquito	*Cx. orientalis*	NS5	MZ868505	
	Sangju-v1	Sangju, ROK	Mosquito	*Cx. orientalis*	E	MZ868506	
	Sangju-v2	Sangju, ROK	Mosquito	*Cx. orientalis*	E	MZ868507	

C, Capsid; *Cx.*, *Culex*; E, envelop; GV, genotype V; NS, non-structural; ROK, Republic of Korea
